# Tick-borne encephalitis: A 43-year summary of epidemiological and clinical data from Latvia (1973 to 2016)

**DOI:** 10.1371/journal.pone.0204844

**Published:** 2018-11-13

**Authors:** Dace Zavadska, Zane Odzelevica, Guntis Karelis, Lelde Liepina, Zane Anna Litauniece, Antra Bormane, Irina Lucenko, Jurijs Perevoscikovs, Linda Bridina, Laura Veide, Angelika Krumina, Jelena Storozenko, Wilhelm Erber, Myint Tin Tin Htar, Heinz-Josef Schmitt

**Affiliations:** 1 Department of Paediatrics, Riga Stradins University, Riga, Latvia; 2 Children's Clinical University Hospital, Riga, Latvia; 3 Faculty of Continuing Education, Riga Stradins University, Riga, Latvia; 4 Department of Infectology and Dermatology, Riga Stradins University, Riga, Latvia; 5 Department of Neurology, Riga East Clinical University Hospital, Riga, Latvia; 6 Department of Neurology, Riga Stradins University, Riga, Latvia; 7 Centre for Disease Prevention and Control of Latvia, Riga, Latvia; 8 National Reference Laboratory, Riga East Clinical University Hospital, Riga, Latvia; 9 Pfizer Vaccines, Medical and Scientific Affairs, Paris, France and Vienna, Austria; University of Minnesota, UNITED STATES

## Abstract

**Background:**

The incidence of tick-borne encephalitis (TBE) varies significantly over time. To better understand the annual incidence of all TBE cases in Latvia we investigated the disease burden in the country from 1973–2016 using several available sources and case definitions.

**Methods:**

We identified cases of TBE from an electronic database (maintained by the Centre for Disease Prevention and Control of Latvia [CDPC]) by the use of ICD-10 diagnosis codes for TBE (A84; A84.0; A84.1; A84.8; A84.9). In addition, previously unreported TBE cases were found by review of TBE diagnoses according to ICD-10 codes in four hospital databases.

**Results:**

From 1973 to 2016 a total of 15,193 TBE cases were reported to the CDPC, 2,819 of which were reported from January 2007 through December 2016, additionally for this time period, 104 cases were identified via hospital survey. From all 2,923 reported cases (2007–2016), 1,973 met TBE case definition criteria and were included in the TBE study analysis. The highest average 10 year incidence was observed from 1990–1999 (27.9 cases per 100,000; range 4.6–53.0), however, the average 10-year incidence from 2007–2016 using officially adopted TBE case definition was 9.6 cases per 100,000 (range 5.8–14.6). For this 10-year time period most cases were adults (95.1%) and male (52.2%). The most common clinical form of TBE was meningitis (90.6%). A tick bite prior to TBE onset was reported in 60.6% of TBE cases and 98.2% of cases were not vaccinated against TBE.

**Conclusion:**

The data demonstrate that the incidence of TBE varies by about one third based on the case definition used. TBE occurs almost entirely in the unvaccinated population. Regular TBE awareness campaigns could encourage the population in Latvia to use protective measures to further control TBE in the country, either via vaccination or tick avoidance.

## Introduction

Tick-Borne Encephalitis (TBE) is a potentially life-threatening infectious disease that occurs in endemic areas across the forested belt throughout Western, Central and Northern Europe through Asia to Hokkaido island in Northern Japan.[1, 2] TBE is caused by the TBE virus (TBEV), a flavivirus which is usually transmitted by infected ticks or in rare circumstances by consumption of unpasteurized dairy products from infected goats, sheep or cows. The main vectors of the TBE virus in Europe are ticks of the Ixodidae family, mainly Ixodes ricinus and Ixodes persulcatus (the latter mainly in Eastern Europe).[3] All three main TBEV subtypes (European, Siberian and Far-Eastern) are carried by ticks in Latvia.[4] Although the disease is preventable by vaccination[5], it continues to be one of the most frequent causes of viral meningitis/encephalitis among both visitors and the local population.[6, 7]

According to published data the number of reported TBE cases in Europe has increased by up to 193.2% in the last 30 years.[7] The availability of improved and cheaper diagnostic tests, increased disease awareness, increased outdoor activities and global warming all have been hypothesized to be contributing to the observed increasing incidence and expanding geographic distribution of the TBEV, which has now become a growing public health concern in many countries.[2, 8] In addition, reliable estimates of TBE incidence are not available in most countries, particularly before 2012. This is largely due to differences in diagnostic criteria, case definitions and valid and consistent reporting systems for TBE infection; all are problems which still exist today. With these methodological limitations in mind, the true and full burden of TBE in Europe remains unclear and likely is/has been underestimated. Therefore, in September 2012 the European Centre for Disease Prevention and Control (E-CDC) included TBE on the list of notifiable diseases in the European Union, and included a case definition for easier and more uniform diagnosis.[9, 10]

Based on national legislation, there has been a countrywide mandatory case-based passive reporting system in place in Latvia since 1973, presided over by The Centre for Disease Prevention and Control (CDPC) of Latvia. However, the E-CDC case definition for TBE was not in use in Latvia until officially adopted in 2012.[9] This definition required 3 criteria: 1) appropriate clinical symptoms (central nervous system [CNS] inflammation); 2) an epidemiological link; and 3) serological confirmation. Aggregated data on TBE cases in Latvia are available from 1955[11], but serological testing for TBE only began in the 1970’s.[12] Before 2012, the reporting of cases in Latvia usually required only a positive serology result, and did not necessarily require symptoms of CNS disease. Thus, reported TBEV infections sometimes included those without evident CNS involvement. Since TBE became notifiable in Latvia, the reported annual number of TBEV-infections (with or without CNS involvement) was surprisingly high. Between 1990 and 2000 Latvia had one of the highest incidences of TBE in the world, ranging from 8 to 53 cases per 100,000 population.[7] With more specific reporting the incidence of TBE has decreased significantly, however Latvia still has extremely high TBE incidence rates compared to other countries in Europe, and has significant differences between regions within the country.

Vaccination remains the most effective protective measure against TBE.[5, 13, 14] The use of anti-TBEV immunoglobulins as post-exposure prophylaxis was abandoned Europe-wide as this method was never proven to be effective in controlled clinical trials. It has been suggested that the application of immunoglobulins may even aggravate the clinical course, although this has never been proven.[15, 16] Current TBE vaccines in use in Latvia include FSME-Immun (Pfizer; formerly TicoVac, used since 1995) and Encepur (GlaxoSmithKline; available since 2001 for adults and since 2002 for children). Both vaccines were found to be highly immunogenic in clinical studies with close to 100% seroconversion rates after the primary vaccine series in both adults and children.[17, 18]

Latvia has had a National TBE Immunization Program since 2006. This program provides TBE vaccines free of charge to children living in highly endemic areas. This program has also covered children without parental care country-wide since 2010. Before the start of the National Immunization Program (between 1997 and 2003) there were some TBE vaccination campaigns for children living in specific highly endemic areas that provided partial reimbursement from the government and various sponsors, but these programs were not implemented on a large scale. Vaccination against TBE is mandatory for those with a high risk of occupational exposure in Latvia, such as forest workers, military personnel, and lab workers. For other residents of Latvia and travelers to the country, vaccination is also strongly recommended, but not reimbursed, although most private insurance companies cover TBE vaccination costs.[19, 20] Unfortunately, the impact of these TBE vaccination programs has not been well documented.

The objective of the current study was to better understand the annual incidence of all TBE cases officially reported to the CDPC of Latvia since the implementation of TBE vaccine and TBE serological testing. The study was also done to specifically investigate the burden and clinical characteristics of TBE in Latvia from 2007 to 2016 using the E-CDC case definition. The results of this study will be the basis for planning and executing a prospective study on the epidemiology of TBE in Latvia and also investigating vaccine effectiveness.

## Materials and methods

### Study design

This was a retrospective database study using officially reported cases of TBE in all age groups, supplemented by a chart review aiming at identifying additional cases for the period 2007–2016 in four most significant treatment institutions in Latvia for TBE cases.

### TBE case definition

For the period from 1973 to 2006 confirmation of TBE infection in Latvia was largely based on detection of anti-TBEV-IgM in blood (often in CSF as well), season, tick exposure and clinical presentation of neurological illness. For nationwide reporting, a TBE case definition has only been used since 2012. However, for the terms of this study, all TBE cases for the current time period (2007–2016) were defined following and adapting the E-CDC definition[9]: 1) Clinical Criteria: Any person with symptoms of inflammation of the central nervous system (e.g. meningitis, meningo-encephalitis, encephalo-myelitis, encephaloradiculitis); 2) epidemiological link as possible exposure to ‘tick bite in an endemic area’, ‘stay in an endemic area’ (applies to all patients hospitalized in Latvia) or ‘alimentary exposure’ and 3) Laboratory Criteria (for Probable case): demonstration of TBE-specific IgM–antibodies in serum sample in a patient with symptoms of inflammation of CNS as mentioned above; For Confirmed case–usually demonstration of TBE specific IgM and IgG antibodies in blood or TBE specific IgM antibodies in CSF in a patient with symptoms of inflammation of CNS.

### Study population

The study population consisted of all TBE cases of any age that had ever been reported to the CDPC of Latvia from 1973 through 2016. Available data from 1973 to 2006 included only the annual number of TBE cases and thus these data could only be used for TBE incidence analyses. This was due to the limitation that serological confirmation may not always have been accomplished and that an unknown fraction of patients may only have suffered from a variety of unspecific symptoms, (e.g. “fever after tick bite”). Case-based data in electronic format were available as of 2007 and thus the main study population for a more detailed analysis was defined as reported TBE cases in Latvia from January 2007 through December 2016.

### Data collection

The CDPC of Latvia provided all TBE cases from their electronic database to our study investigators at the Riga Stradins University categorized by ICD-10 diagnosis codes for TBE (A84; A84.0; A84.1; A84.8; A84.9). Hospitals across Latvia were contacted and asked to provide the respective patient’s case histories/medical records for additional clinical data. Data collection from patient records was guided by a questionnaire that included information about: age, gender, region of residence, vaccination history (including number of doses received and the vaccination date of the most recent dose), tick bite history, exposure to the same identified infected food source as a confirmed TBE case during an outbreak, clinical symptoms, laboratory test results, lumbar puncture results, duration of hospitalization and clinical outcome at discharge (TBE form). All patient data in data collection process were de-identified.

In total, 26 Latvian hospitals provided TBE case medical records for chart review; one hospital did not share TBE case medical records. In addition, previously unreported TBE cases for the time period 2007–2016 were found by review of TBE diagnoses according to ICD-10 codes in four hospital databases. All four hospitals are located in central region of Latvia, Riga. They include country’s most important multi-profile treatment institution for children–Children’s Clinical University Hospital, for adults–Riga East Clinical University Hospital and Pauls Stradins Clinical University Hospital and the only specialized treatment institution in Latvia in the field of infections–Latvian Centre of Infectious Diseases. These cases were also included in the study population for TBE analysis. Missing or negative results of TBE serology were double checked at the National Reference Laboratory.

### Statistical methodology and analysis

TBE incidence rates from 1973 to 2016 were calculated as the number of cases per 100,000 individuals of the Latvian population by age groups. Population statistics were based on data from the Central Statistical Bureau of Latvia.

For data collected from 2007–2016 descriptive analyses such as frequency distributions, percentages, medians and ranges were used to summarize the data (annual incidence of TBE for each year, age groups, gender, immunization status, region of Latvia, clinical outcome of the cases etc.). Incidence rates were also summarized by year and age group.

Subjects of any age were included and categorized into two main groups: children (<18 years), and adults (>18 years). More detailed clinical data analyses were performed in three age groups (<18 years; 18-<60 years; >60 years).

For the period from 2007 to 2016, cases of TBE without serological confirmation (negative IgM antibodies or missing test results) were excluded from the study. Also patients with confirmed TBEV infection (i.e. detection of specific antibodies to the TBEV) presenting to hospitals with just fever and/or other unspecific possible symptoms of a TBEV-infection but with no symptoms indicating disease of the CNS (“fever forms”) were identified but excluded from the current TBE case analysis. However, considering the high prevalence of TBEV infections among the Latvian population, these cases were collected separately and additional analyses were performed to understand why these cases, despite lack of CNS-symptoms, were initially considered as TBE and serologically tested.

### Definitions

Vaccine failure–TBE in an individual hospitalized (1) at least 2 weeks after the second dose but before 12 months before the third dose; (2) after the third dose within 3 years before the first booster; (3) within 5 years after the first of the following booster doses (patients >60 years) or within 3 years after the first or following boosters in patients age >60 years.

Vaccine ineffectiveness—TBE in an individual who had received an incomplete vaccination series, specifically (1) only a first dose; (2) only 2 doses and hospitalized less than 2 weeks or after 12 months after the second dose; (3) the third dose more than 3 years before the first booster; or after 5 years from the first of the following booster doses (patients >60 years) or after 3 years from the first or following boosters in patients age >60 years.

### Ethical conduct of the study

The study was conducted in full compliance with any legal and regulatory requirements. Ethical approval of this study was determined by Riga Stradiņš University Ethical Commissions (September 8, 2016, Nr. 24/08.09.2016.).

## Results

### Epidemiological data

**Fig 1:**
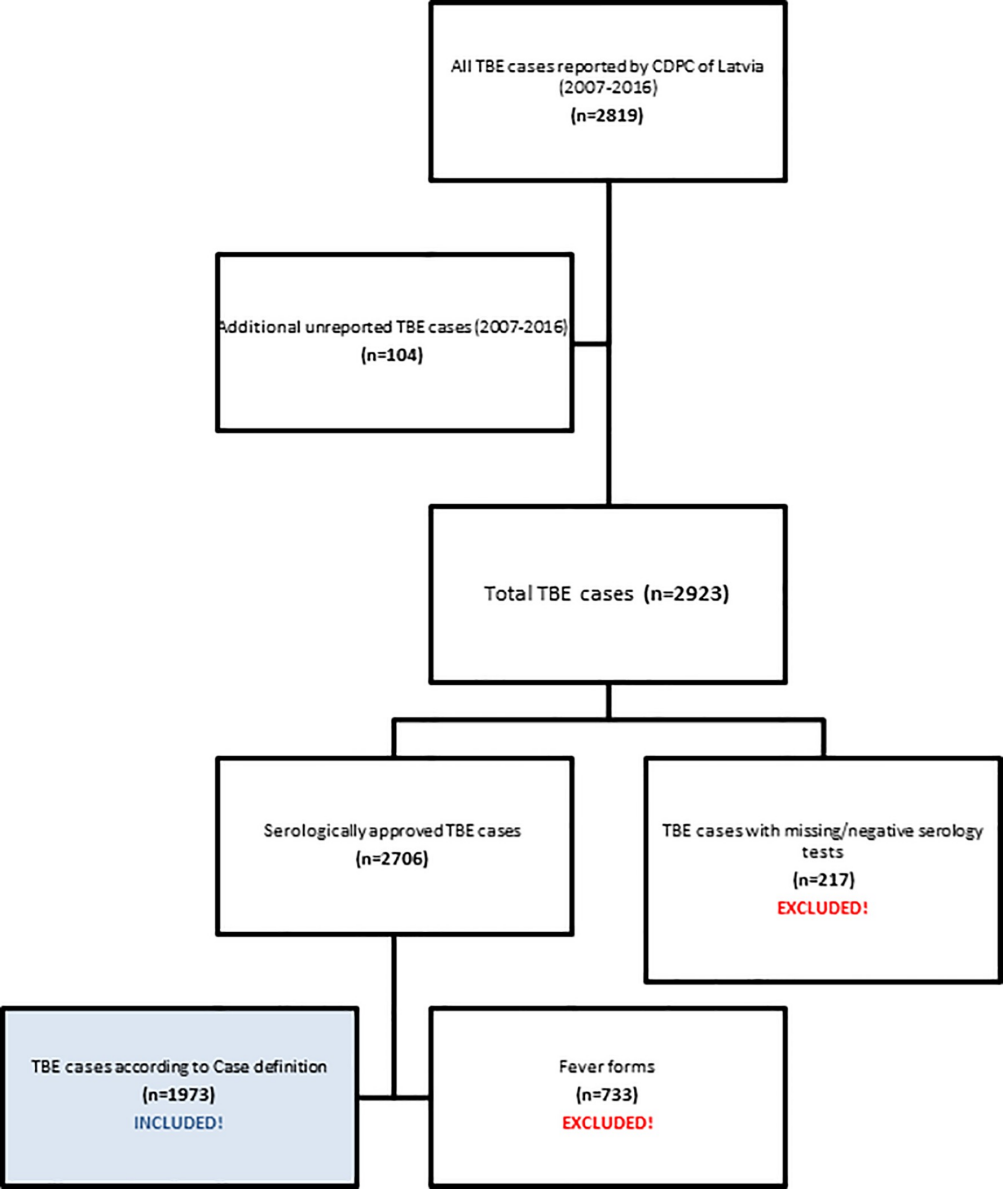
Flow chart of TBE patients selected to the study.

The total population of Latvia is estimated to be roughly 2 million people (2.416 million in 1973; 1.96 million in 2016).[21, 22] From 1973 through 2016 a total number of 15,193 TBE cases were reported to the CDPC of Latvia, 2,819 of which were reported from January 2007 through December 2016 (Fig 1), additionally for this time period, a review of four hospital databases for TBE identified an additional 104 cases that had not been reported to the CDPC previously (104/2,923 = 3.6%). From all 2,923 reported cases, only 1,973 TBE cases (2007–2016) met TBE case definition criteria (CNS inflammation signs plus serological confirmation; as Probable case) and were included in the TBE study analysis. The remaining 950 cases (32%) were excluded from current analysis and were largely from patients hospitalized with serological confirmation of a TBEV infection but without any clinical signs of CNS disease (n = 733; 25%) and with negative/missing serological data (n = 217; 7%).

**Fig 2:**
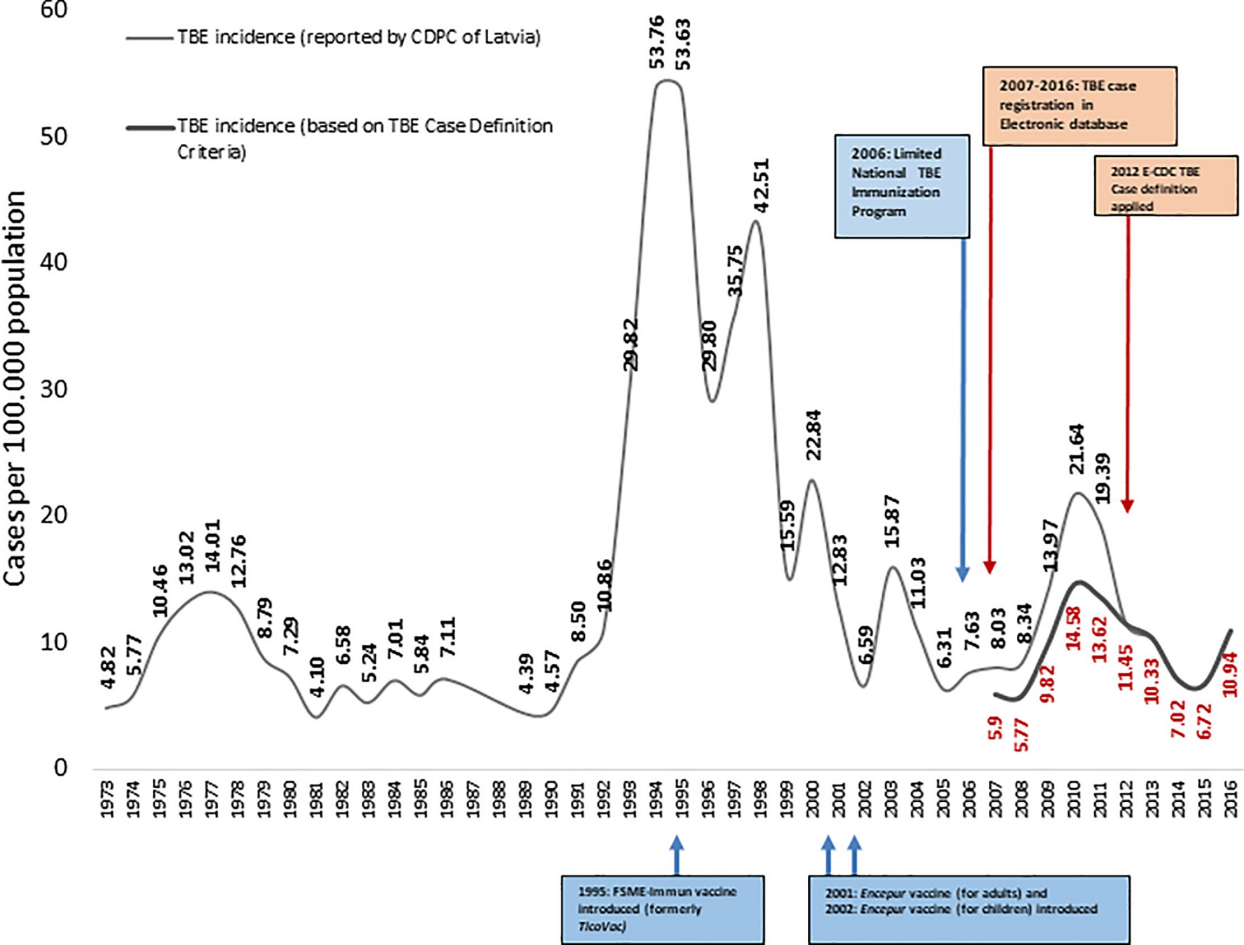
TBE incidence rates in whole population; 1973–2016; vaccine uptake gradually increased from 39% (2009) to 52% in 2015.

Annual TBE incidence rates for the 1973–2016 timeframe are displayed in Fig 2. The highest average 10 year incidence was observed from 1990–1999 (27.9 cases per 100,000 population; range 4.6–53.0) and the average 10-year incidence of TBE from 2007–2016 using the TBE case definition was 9.6 cases per 100,000 population (range 5.8–14.6). The officially reported incidence of TBE after implementation of the E-CDC case definition decreased by about 40% (2011: 19.4 cases per 100,000; 2012: 11.5 cases per 100,000). The highest yearly incidence after introduction was observed in 2010 (14.6 cases per 100,000), followed by significant decreases in 2014 (7.0 cases per 100,000; p<0.001) and 2015 (6.7 cases per 100,000). While the annual incidence shows a general tendency to decrease, an increase was observed in 2016 (10.9 cases per 100,000) compared to the prior year.

**Fig 3:**
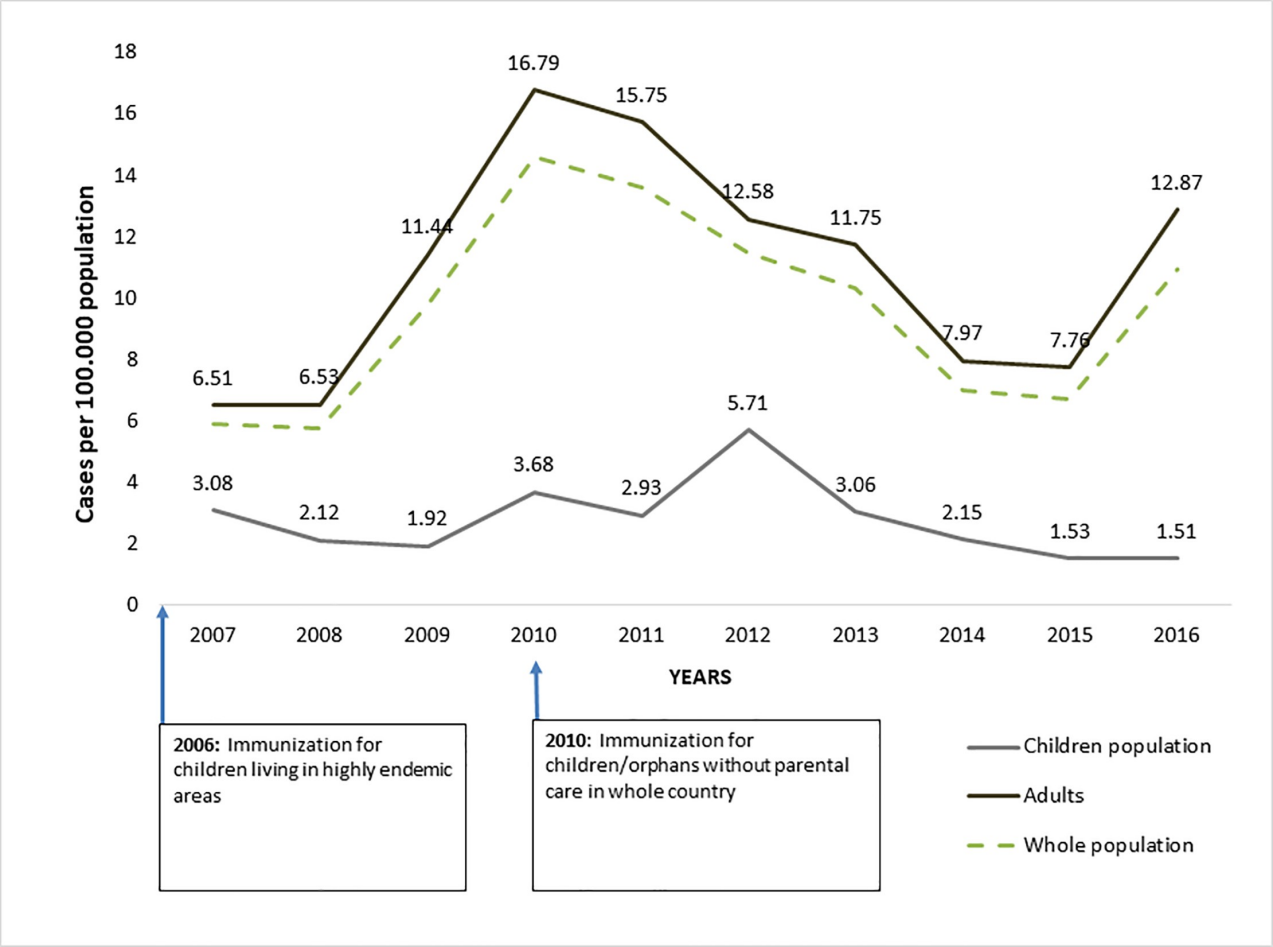
TBE incidences in children (<18 years) and adults, 2007–2016, using the E-CDC case definition.

Pediatric TBE incidence rates between 2007 and 2016 were lower than that of the overall population (Fig 3). In the last decade, the highest TBE incidence in children was observed in 2012 (5.7 cases per 100,000). This was followed by an almost four fold decrease by 2016 (1.5 cases per 100,000).

**Fig 4:**
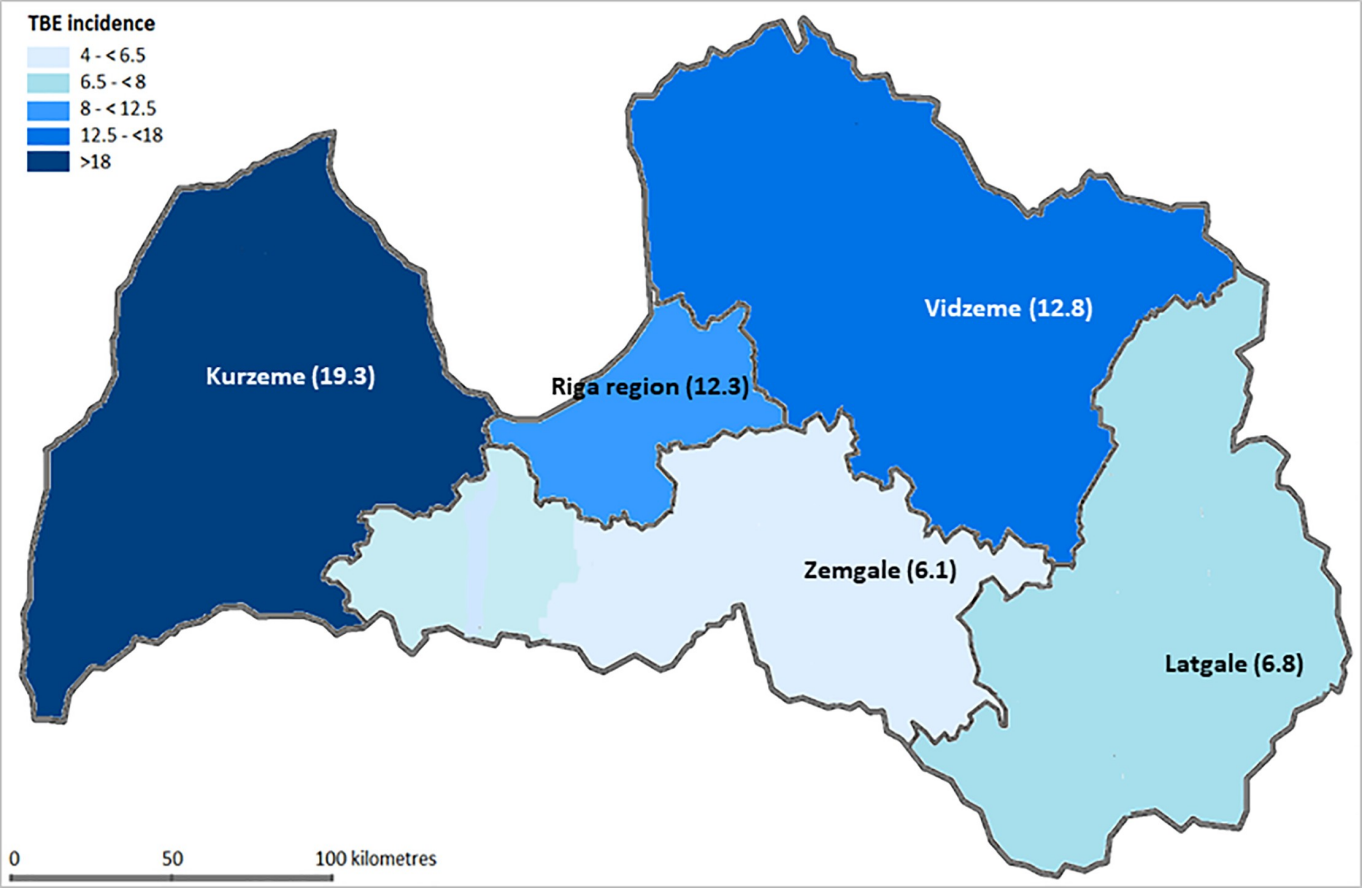
TBE average incidence rate per 100 000 inhabitants by regions of Latvia (period 2007–2016).

TBE incidence from 2007 to 2016 was found to vary between different regions of Latvia (Fig 4). The highest incidence of TBE over this time was observed in Kurzeme (19.3 cases per 100,000) and Vidzeme (12.8 cases per 100,000). The lowest incidence was reported from Latgale (6.8 cases per 100,000) and Zemgale (6.1 cases per 100,000).

**Fig 5:**
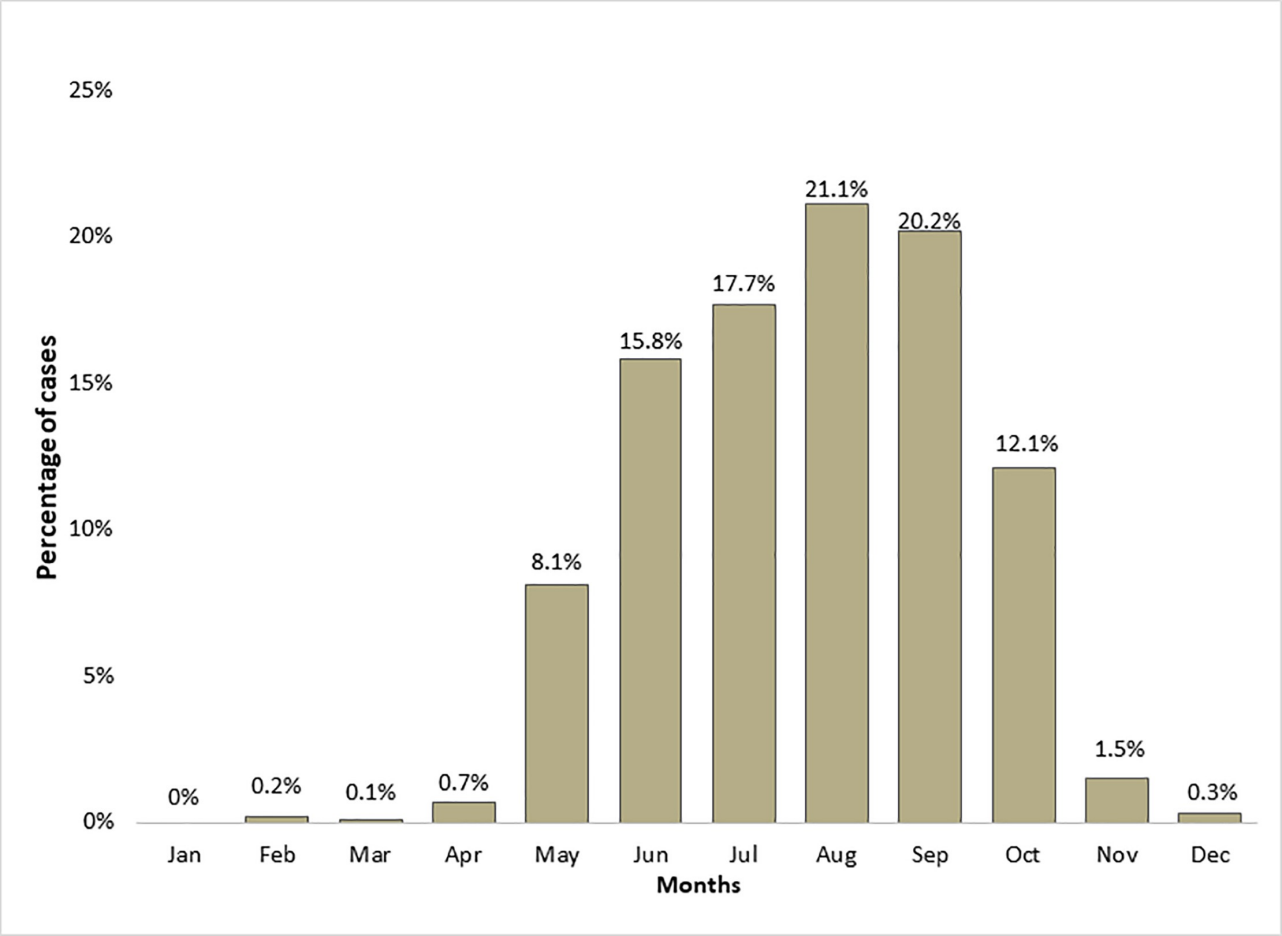
Average TBE seasonality by months (period 2007–2016;n = 1929).

Data concerning the onset of TBE country-wide by month were available for 97.8% (n = 1929) of patients. Most cases were reported in summer months (July: 350 cases [17.7%]; August: 417 cases [21.1%]; September: 398 cases [20.2%]) (Fig 5). In addition, there were outlying cases reported during the winter with 6 cases in December (0.3%), 4 cases in February (0.2%) and 1 case in March (0.1%).

### Patient characteristics

**Fig 6:**
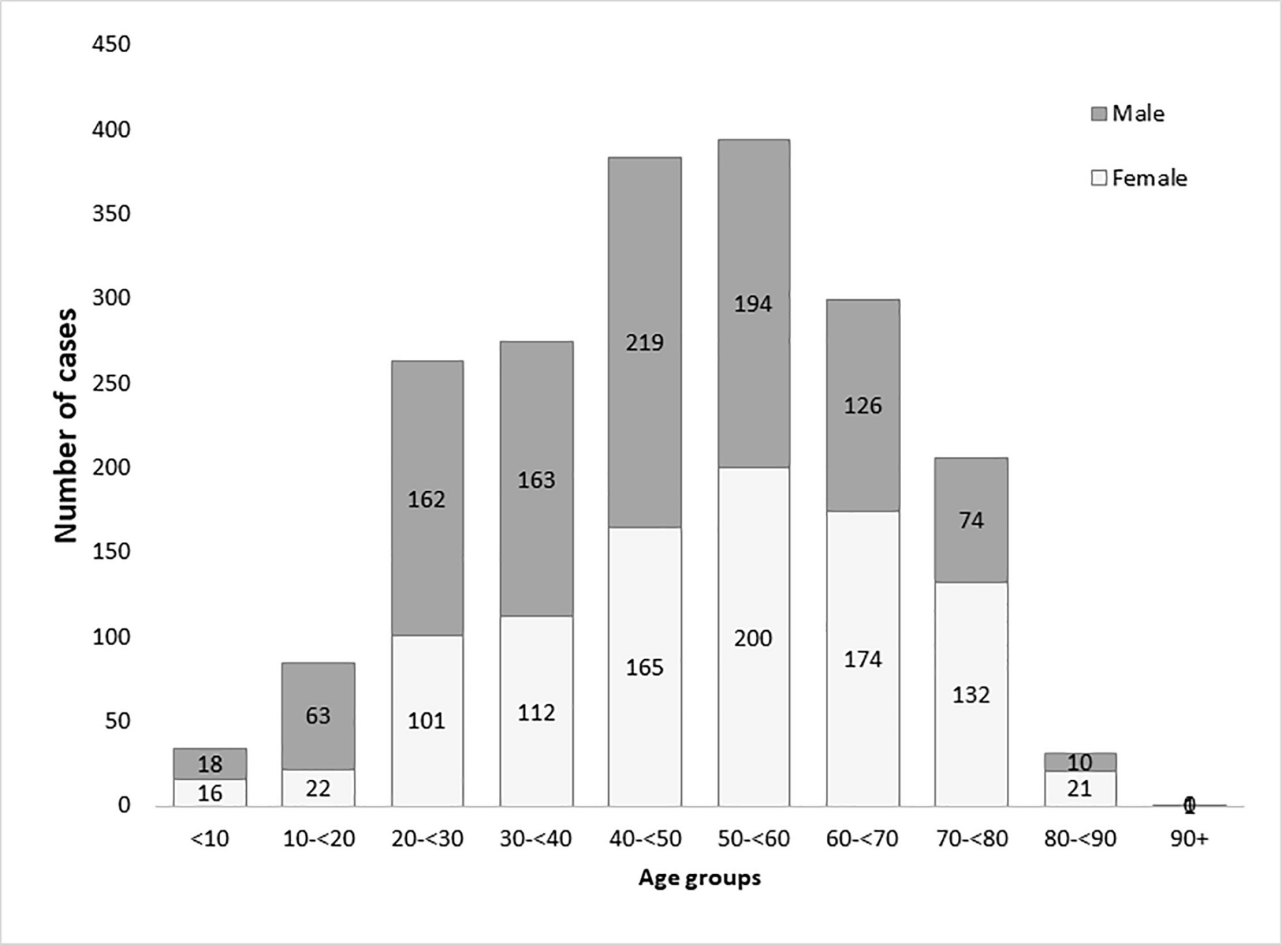
Number of TBE cases per age group and gender.

A total 1,973 serologically confirmed TBE cases were reported between 2007 and 2016 according to the TBE case definition and were analysed for clinical characteristics. Most of the cases were adults (95.1%; n = 1,877) (Fig 6), and 52.2% of cases were seen in males (n = 1,029). The median age in the adult population was 49 years (range: 18–91), and the median age in children was 11.5 years (range: 2–17). There were no registered TBE cases in children younger than 2 years.

**Fig 7:**
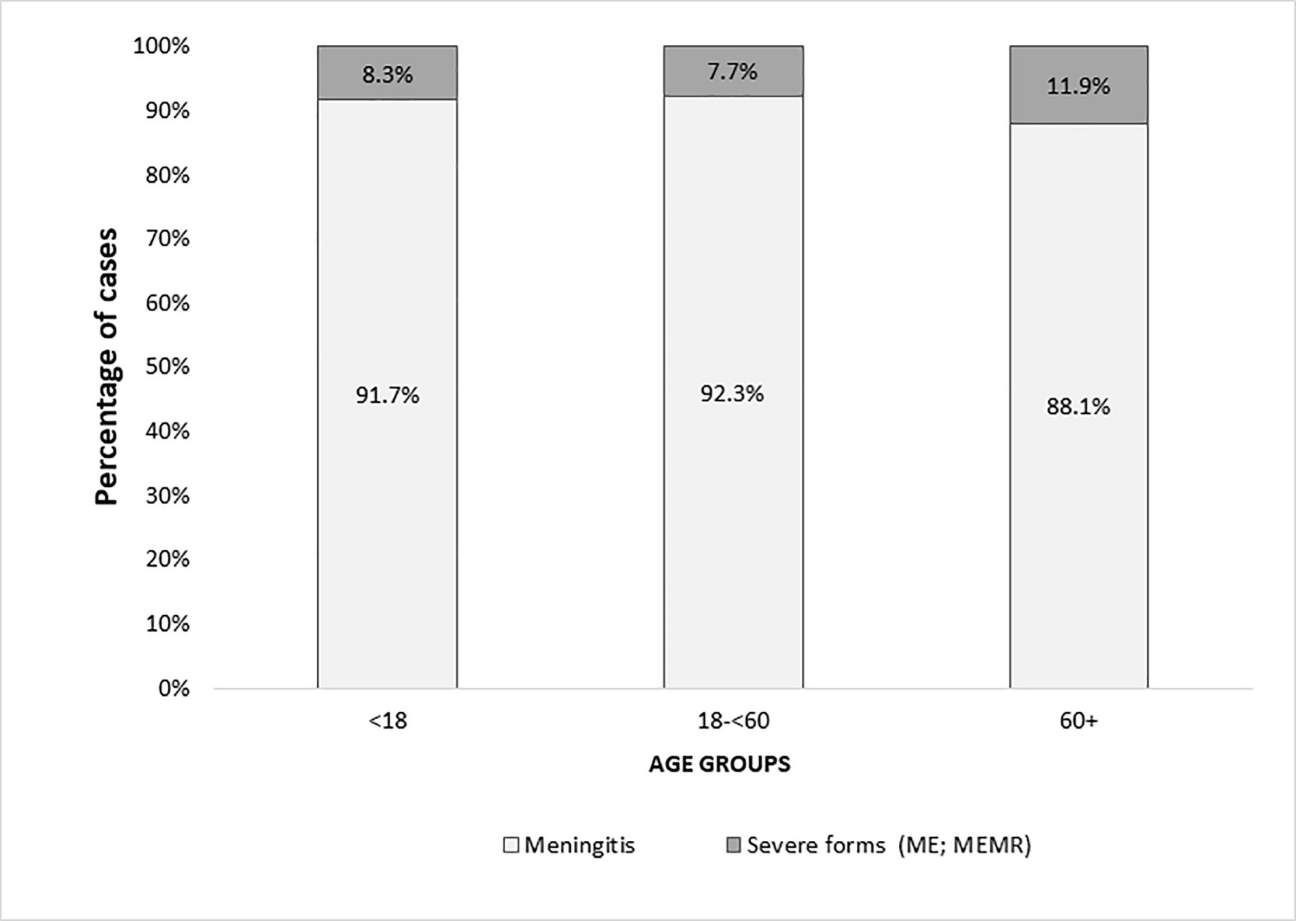
TBE clinical forms by age groups.

The most common clinical form of TBE for children and adults was meningitis (90.6%; n = 1,787) (Fig 7). Severe forms like meningo-encephalitis and meningo-encephalo-myelo-radiculitis were seen in 8.9% of patients (n = 174). In 0.6% of cases (n = 12), CNS inflammation signs were present with unknown clinical form. The median duration of hospitalization was 12 days (range: 1–221 days). A tick bite prior to TBE onset was reported in 60.6% of TBE cases (n = 1,139). Transmission via unpasteurised goat’s milk was considered a possible route of TBE transmission in 0.7% of cases (n = 14). Lumbar puncture was done in 85.3% (n = 1,604) of all TBE cases. CSF pleocytosis (more than 5x106 leukocytes per litre) was observed in 97.6% (n = 1,563) of all tested patients.

### Immunization status

According to hospital records, 1.6% (n = 32) patients had been vaccinated against TBE with at least one dose. 98.2% (n = 1,937) of patients (2007–2016) were not vaccinated against TBE. Vaccination status was unknown for 4 (0.2%) patients. Overall, TBE cases were admitted to hospital a median of 1,051.5 days (range: 8–3,345) after the last TBE vaccine dose administered (Table 1).

**Table 1:** Number of vaccine doses administered and median interval of TBE occurrence in any patient with TBE who had received at least one TBE vaccine dose.

Doses of TBE vaccine administered before hospitalization for TBE	Number of cases; (%)	Median interval from last vaccine dose to hospitalization with TBE
1^st^ dose	3; (9.4%)	3 years (range 2 to 4 years)
2^nd^ dose	9; (28.1%)	2 years (range 5 days to 5 years)
3^rd^ dose	9; (28.1%)	3 years (range 1 to 9 years)
4^th^ dose (1^st^ booster)	6; (18.8%)	2 years (range 2 to 9 years)
5^th^ dose (2^nd^ booster)	1; (3.1%)	5 months
6 and more doses	4; (12.5%)	2 years (range 8 months to 3 years)

TBE following anti-TBE vaccination occurred in 18 patients after exclusive use of FSME IMMUN (TicoVac, Baxter; now Pfizer)(56.3% of total vaccinated TBE cases), and 9 patients after exclusive use of Encepur (Novartis; now GSK)(28.1% of total vaccinated TBE cases). Five patients who contracted TBE (15.6% of total vaccinated TBE cases) had received mixed vaccination schedules using both Encepur and FSME IMMUN.

**Fig 8:**
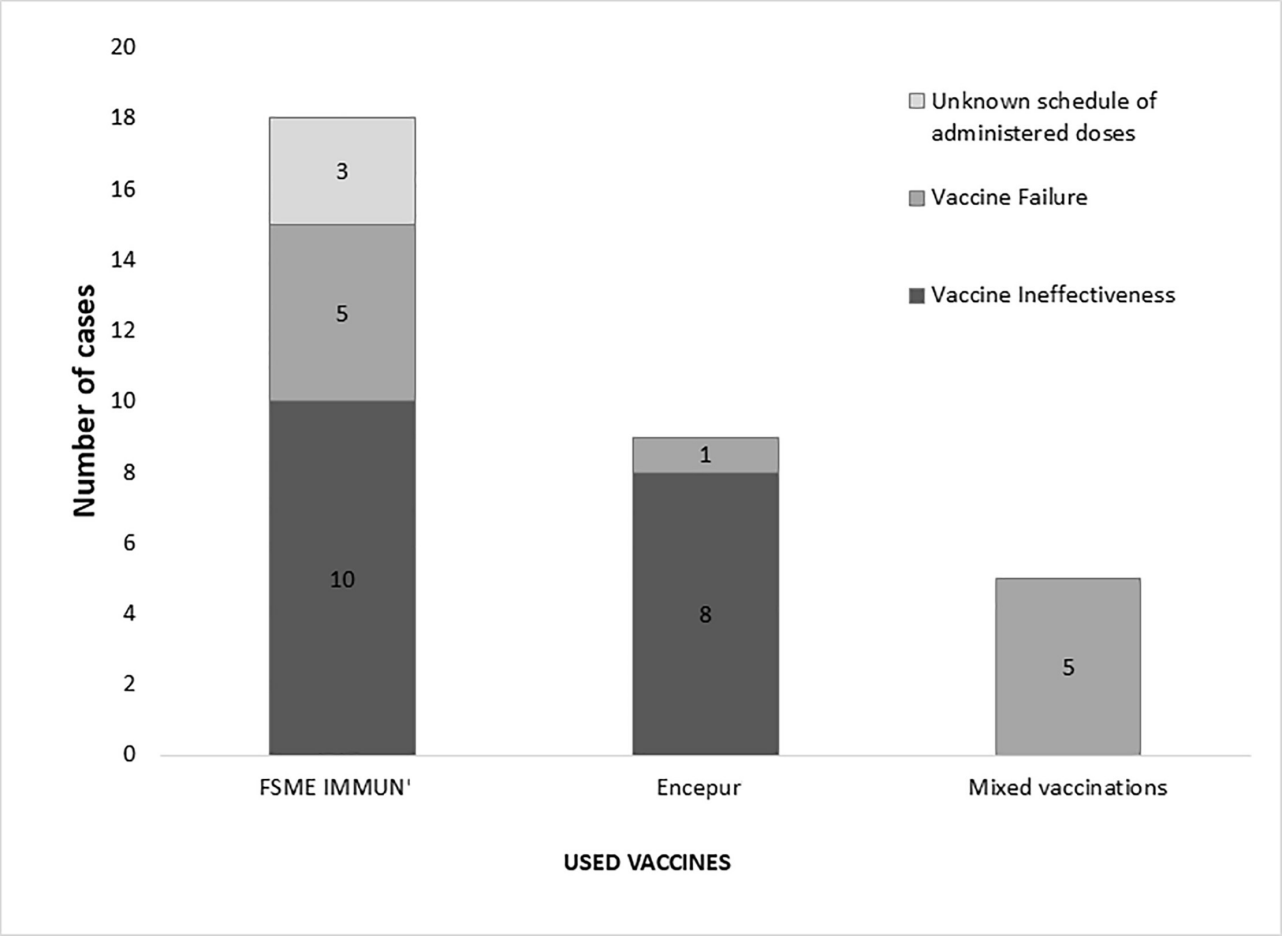
Vaccine outcome in previously vaccinated TBE cases.

Vaccine failures were identified in 34.4% (n = 11) of those TBE cases that had previously been vaccinated (Fig 8, Table 2). Thus, approximately, 1.1 TBE vaccine failures cases occurred annually over the 10 year period from 2007 to 2016. Vaccine failure cases were observed in all age groups: 2 in those aged <18 years (50%), 6 in those aged 18-<60 years (26.1%) and 3 in those aged >60 years (60.0%). A total of 5 vaccine failures were seen in patients solely vaccinated with FSME IMMUN, 1 vaccine failure occurred in a patient solely vaccinated with Encepur and 5 vaccine failures were observed in patients who had received a mixed vaccination schedule including both vaccines. Vaccine ineffectiveness was seen in 10 TBE cases previously vaccinated with FSME IMMUN and in 8 TBE cases previously vaccinated with Encepur. The exact immunization schedule remained unknown in 3 TBE cases previously vaccinated with FSME IMMUN.

**Table 2:** Number of “vaccine failures” and “vaccine ineffectiveness” cases of TBE by vaccine doses and product used.

	*FSME IMMUN*		*Encepur*		*Mixed vaccination*
	Vaccine failure	Vaccine ineffectiveness	Vaccine failure	Vaccine ineffectiveness	Vaccine failure
After 1^st^ dose	-	2 cases	-	1 case	-
After 2^nd^ dose	1 case	4 cases	-	4 cases	-
After 3^rd^ dose	1 case	3 cases	-	3 cases	-
After 4^th^ dose (1^st^ booster)	1 case	1 case	-	-	3 cases
After 5^th^ dose (2^nd^ booster)	1 case	-	-	-	-
After 6 and more doses	1 case	-	1 case	-	2 cases

### TBE infections (2007–2016) that did not meet ECDC-TBE case definition criteria

There were 733 reports of TBE infection between 2007 and 2016 that did not meet the E-CDC case definition. Such infections represented 27.1% of all serologically confirmed cases of TBEV infections reported to CDPC between 2007 and 2016. All these patients had fever (“fever form”), no CNS inflammation signs clinically. Tick bite prior to the onset of symptoms was noticed in 52.1% of these cases (n = 382), and transmission via unpasteurised goat milk was considered a possible source of (the asymptomatic) TBE infection in 2.7% of cases (n = 20). Most of the cases occurred during periods of high tick activity; July (21.0%; n = 154), August (20.3%; n = 149) and September (16.5%; n = 121).

From all cases initially reported to CDPC, 7.5% (n = 217) did not meet the laboratory case definition criteria: in 215 cases serology was negative, nevertheless, repeated testing and test results remained unknown for 2 cases. None of these 217 cases were included in the current analysis.

## Discussion

Since TBE became notifiable in Latvia the highest average 10 year incidence was observed between 1990 and 1999 (27.9 cases per 100,000) (Fig 2). There is no single causal factor that alone can explain this peak, but rather a complex mix of several factors. One factor that likely contributed to the peak was high tick activity that may (at least in part) have resulted from largely abandoning the use of pesticides on farmlands and meadows.[11] Another likely related factor was an increased exposure to ticks as a result of increased outdoor economic activities in the wake of the political and socio-economic changes of 1989, nicely summarized by Sumilo et al.[8] Moreover, with the increased use of serological confirmation of TBE infections, there may have been increased awareness (and fear) of TBE, resulting in more frequent testing and the detection of more cases. It remains unknown which proportion of these cases had CNS involvement. One may speculate from the dataset provided here for the period between 2007 and 2016 that about one third of cases between 1990 and 1999 represented “fever forms”, resulting in a peak TBE incidence of about 36 cases per 100,000. Still, this incidence is among the highest in the world, and if accurate, may be a consequence of increased outdoor exposure as discussed elsewhere.[8, 12]

The adoption of the E-CDC case definition for TBE in Latvia in 2012 ensured a specific assessment of the burden of disease due to TBE and the impact of vaccination. Specifically, the reported annual TBE incidence markedly decreased by about 40% from 19.4 cases per 100,000 in 2011 to 11.5 cases per 100,000 in 2012, just one year after the new case definition had been implemented. Taking away the fever-only cases from 2011, the incidence drops to 13.6 in the same year. Comparing TBE incidences for longer periods before (2007–2011) and after (2012–2016) the E-CDC case definition was used in Latvia incidence still dropped by about one third (from 14.3 to 9.3 cases per 100,000) and was largely comprised of”fever form” cases.

Looking at Fig 2, in the past 7 years the annual incidence of TBE continued to decrease from 14.6 cases per 100,000 in 2010 to 6.7 cases per 100,000 in 2015. However, in 2016 it increased again by about one third to 10.9 cases per 100,000 due to an increased number of TBE cases among the adult population. Heinz et al. suggested that long range-TBE incidences peak in >5 year cycles.[13] The TBE increase in adults seen in Latvia in 2016 may be the beginning of such a new cycle, particularly in a largely unvaccinated population. The explanation for generally decreasing TBE incidence in Latvia in recent years could be the increasing vaccine uptake due to The National TBE Immunization Program. This program resulted in an increase in vaccine uptake from 39% in 20095 to 52.5% by 2015.[23] Moreover, TBE vaccine uptake in children reached 22% nationwide, and as high as 77% in highly endemic areas. This likely played a role in reducing the proportion of TBE cases among children from 12.5% in 2001 to 3.6% in 2010[7]. Market research data from 2015 shows that FSME-Immun is the most commonly used TBE vaccine in Latvia with up to 86% market share among those vaccinees who received at least one dose of TBE vaccine and who knew which vaccine they had received.[24]

TBE incidence rates in Latvia also varied by geographic area during the years investigated. This can perhaps be explained by different factors influencing tick activity/density and degree of human contact with the tick habitat such as climatic conditions (a gradual decrease of the influence of oceanic air masses towards the east of country)[25], vegetation peculiarities (proportion of used or abandoned agriculture fields, bushlands, forest types and forest clearances), and population density differences and human lifestyle modifications. Climatic factors and human behaviour may also play a role, as warmer summer months may result in more tick exposure due to increased outdoor activities (collection of mushrooms and berries, etc.).[26] In addition, epidemiological investigations suggest that 20%-40% of ticks in Latvia were infected with TBEV, and that ticks in Latvia carried a higher TBEV load than in other endemic countries.[7] However, a recent study revealed comparatively low TBEV loads in Latvian ticks during 2014 (3%) and 2015 (2.1%).[27]

The most common clinical manifestation of TBE reported in all age groups was meningitis. Meningitis is the mildest and most common TBE neurological form also comparing to the literature data.[7] Most likely also TBE awareness campaigns and society education plays an important role in early recognition of disease symptoms, therefore patients present more timely with milder neurological involvement and receive an adequate treatment avoiding further disease development. In adults, the more severe TBE forms (meningo-encephalitis, meningo-encephalo-myelo-radiculitis) were reported somewhat more frequently among patients >60 years (7.7% in adults 18-<60 years versus 11.9% in those >60 years; p = 0.0012). It is worth considering that the growing population of “healthy and active elderly” as a relevant target for vaccination in order to avoid these severe cases. Additionally, this age group faces the risk of becoming seronegative earlier after vaccination.[16] In the paediatric population severe forms were reported in 8.3% of cases. Nonetheless, in children TBEV infections may more frequently cause nonspecific changes that are clinically significant for the individual and that may result in long-term-sequelae[28], but which are not detected by the current standard TBE case definition for meningitis/encephalitis as applied here. Although “fever forms” did not meet E-CDC TBE case definition criteria, additional analysis showed that they were presented in almost one third of hospitalized patients. However, real estimate of asymptomatic TBE infections (“fever forms”) cannot be obtained from this study, because only hospital data were analyzed and patients without neurological symptoms most likely do not require hospitalization.

The data shown here demonstrate that TBE occurs almost entirely in the unvaccinated population: 98.2% of cases in this analysis were not vaccinated against TBE. As expected, true vaccine failure cases did occur, however at a relatively low estimate of 1.1 cases annually among the total vaccinated population. Further prospective studies are necessary to collect more precise data about these possible vaccine breakthrough cases and vaccine effectiveness. Notably, a tick bite prior to the onset of TBE was noticed by just 60.6% of TBE cases. This is surprising, given Latvia is a country with an assumed high awareness of TBE. Thus, clinicians must not rule out TBE in patients with CNS disease based on the lack of remembering a tick bite in an endemic area. Considering this, and following the Austrian example, a Latvian national TBE immunization program with partial reimbursement and annual vaccination reminder campaigns may result in a vaccine uptake of >80%. Such a program would reduce the disease burden in Latvia log fold.

An important limitation of this study was that all included TBE cases were defined as “probable cases” according to E-CDC case definitions based on positive TBEV specific IgM antibodies in serum samples. The lack of retrospective data for other laboratory tests limits the definition of included TBE cases to “confirmed cases” according to laboratory criteria. Lumbar puncture was not done in all TBE cases presenting with neurological signs (headache, fever, positive meningeal symptoms, paresis etc). CSF pleocytosis is a laboratory sign of CNS inflammation, however as this study was retrospective in nature, in some cases clinical criteria for the diagnosis of TBE was met based only on the typical symptoms of CNS involvement. In some cases lumbar puncture was done with normal findings, despite the patient having clinical signs of CNS inflammation. Such confirmed TBE cases with severe neurological involvement, but without CSF pleocytosis have been described previously.[29] Another limitation to the study was that patients presenting with “fever form” (lack of signs of CNS inflammation), were not analyzed further. Patients presenting with fever could have displayed signs of CNS inflammation and met case definitions for TBE if lumbar puncture was performed. In comparison to previous literature, we found a higher proportion of cases with meningitis versus encephalitis. As our study was population based with detailed review of hospital charts, this finding could be due to reporting bias with respect to more severe cases. In other studies with more severe cases, encephalitis or meningoencephalitis may be over-represented. As there is a very high awareness of TBE in Latvia, more testing may be done even in milder forms of the disease. In addition, there could be differences in TBE virus subtypes or even more virulent strains occurring in Latvia and the Baltics as compared to other parts of Europe. We are currently conducting a prospective study to confirm our study’s findings.

In summary, the incidence of TBE in Latvia varies annually and is very difficult to predict. The data presented here suggest that the national TBE immunization program in Latvia may have already had a detectable impact with case numbers declining, as particularly documented in children. Vaccination is a very effective prophylaxis against TBE, and immunization against TBE has been shown to be cost-effective in other European countries like Austria and Slovenia.[30, 31] The current TBE case definition and the need to report TBE cases should be reinforced among physicians and health care workers alike in order to maintain the validity of the reported burden of disease in Latvia. In addition, regular TBE awareness campaigns could encourage the population in Latvia to use protective measures to further control the TBE in the country, whether they be specific (vaccination) or non-specific (tick-human contact avoidance etc.) in nature.
